# Metabolic Response of the Yeast *Candida utilis* During Enrichment in Selenium

**DOI:** 10.3390/ijms21155287

**Published:** 2020-07-25

**Authors:** Marek Kieliszek, Katarzyna Bierla, Javier Jiménez-Lamana, Anna Maria Kot, Jaime Alcántara-Durán, Kamil Piwowarek, Stanisław Błażejak, Joanna Szpunar

**Affiliations:** 1 Department of Food Biotechnology and Microbiology, Institute of Food Sciences, Warsaw University of Life Sciences—SGGW, Nowoursynowska 159 C, 02-776 Warsaw, Poland; anna_kot@sggw.edu.pl (A.M.K.); kamil_piwowarek@sggw.edu.pl (K.P.); stanislaw_blazejak@sggw.edu.pl (S.B.); 2 Institute of Analytical Sciences, IPREM, UMR 5254, CNRS-UPPA, Hélioparc, 2 Avenue Angot, 64053 Pau, France; katarzyna.bierla@univ-pau.fr (K.B.); j.jimenez-lamana@univ-pau.fr (J.J.-L.); 3 Analytical Chemistry Research Group, Department of Physical and Analytical Chemistry, University of Jaen, 23071 Jaen, Spain; jaduran@ujaen.es

**Keywords:** selenium, *Candida*, yeast, antioxidant enzymes, selenium speciation

## Abstract

Selenium (Se) was found to inhibit the growth of the yeast *Candida utilis* ATCC 9950. Cells cultured in 30 mg selenite/L supplemented medium could bind 1368 µg Se/g of dry weight in their structures. Increased accumulation of trehalose and glycogen was observed, which indicated cell response to stress conditions. The activity of antioxidative enzymes (glutathione peroxidase, glutathione reductase, thioredoxin reductase, and glutathione S-transferase) was significantly higher than that of the control without Se addition. Most Se was bound to water-insoluble protein fraction; in addition, the yeast produced 20–30 nm Se nanoparticles (SeNPs). Part of Se was metabolized to selenomethionine (10%) and selenocysteine (20%). The HPLC-ESI-Orbitrap MS analysis showed the presence of five Se compounds combined with glutathione in the yeast. The obtained results form the basis for further research on the mechanisms of Se metabolism in yeast cells.

## 1. Introduction

Selenium (Se) is an important element involved in many metabolic processes [1]. It has been recognized as an essential nutrient for its antioxidant properties [2], and it plays an important role in thyroid hormone metabolism and immune defense mechanisms [3]. Se deficiency is associated with many severe cardiovascular diseases [4,5] and increased susceptibility to viral or bacterial infections [6]. The gap between the essentiality and the toxicity of Se is particularly narrow and depends on the chemical form [7].

Inorganic Se found in soil is metabolized by plants and microorganisms to organic forms that can be eliminated by volatilization [8] or bioaccumulated in forms that are more accessible to animals and humans [9]. The metabolism of Se in some plants is induced in the context of bioremediation and as a source of phytochemicals for new potential therapeutic agents. The principal chemical forms of Se in plants include selenomethionine (SeMet), methyselenocysteine, and glutamyl-selenomethyl-selenocysteine [8].

Yeast fermented in Se-rich media has been studied for a long time as the best source of Se for food and feed supplements [10,11]. The most popular yeast *Saccharomyces cerevisiae* grown in the presence of selenite and/or selenate can accumulate up to 3000 mg Se/kg [10]. The total SeMet concentration of >60% has been considered as a measure of: (1) the “organic” characteristic of the Se-rich yeast; (2) the efficiency of the biotechnological enrichment process; and (3) the product quality [12]. Other yeast varieties such as *Torula* (*Candida utilis),* which is widely used as a “natural” flavoring agent in processed foods and pet foods for replacing the flavor enhancer monosodium glutamate [13], may not share this metabolomics pathway. Indeed, Se-rich *Torula* showed low (<10%) concentration of SeMet but high (>80%) concentration of selenohomolanthionine [14]. The ability of Se to bind to the biomass of *S. cerevisiae* ATCC MYA-2200 and *Candida utilis* ATCC 9950 was compared, and it was observed that yeasts of the genus *Candida* were more efficient in binding Se [15]. Effects of Se on morphological changes in cells of the yeast *Candida utilis* were studied [16]. The understanding of the metabolic processes is crucial for the control of Se-enrichment biotechnologies.

High concentrations of Se are a factor of stress for yeast. Se inhibits cell proliferation through cell cycle progression, which leads to a reduction in the potential of lipid and mitochondrial membranes and eventually results in cell death [17]. Under the appropriate growth conditions, yeasts can accumulate Se in their cellular structures and transform them into various chemical forms. The common parameters investigated include: (i) the activity of antioxidant enzymes, which ensures proper protection of metabolic processes as well as the functioning of the entire metabolic system in yeast cells; and (ii) the synthesis of carbohydrates, namely trehalose and glycogen. The function of trehalose is to protect and stabilize membranes, which prevents the loss of cellular components. Trehalose protects lipids against oxidation [18]. The glycogen content in yeast is related to the growth rate of microorganisms [19]. Yeast can accumulate high levels of glycogen in order to survive stress conditions caused by various environmental factors (including the presence of Se) [20]. The development of large-scale metabolomics tools based on mass spectrometry allows monitoring hundreds of Se compounds involved in the metabolomics pathways [21]. The interaction between Se metabolic pathways and the activity of the antioxidant system is very complicated, and only a systematic approach would allow analyzing their interrelationships. Establishing a network of interactions between these routes is a challenge.

The present study aimed to determine the effect of Se on the growth of the yeast *C. utilis* ATCC 9950. The metabolic response of *C. utilis* during enrichment in Se, including oxidative stress markers (trehalose and glycogen), was examined, together with the activity of individual antioxidative enzymes and speciation of Se in Se-enriched yeast biomass. The presence of elemental Se in the cells was also determined. To the best of our knowledge, this is the first attempt to probe the effect of Se on the content of carbohydrates and formation of Se-nanoparticles (SeNPs) in *C. utilis*.

## 2. Results and Discussion

### 2.1. Effect of Se on Yeast Growth

*Candida utilis* ATCC 9950 *C. utilis* ATCC 9950 was able to grow in control and Se-supplemented media. After introducing the yeast inoculum into the control medium without the addition of Se, the optical density (OD) was approximately 0.2. In the Se medium, the yeast had an optical density of 0.3. The yeast grown in medium without Se addition showed higher OD values on the obtained growth curves in all time variants. The study showed that the concentration of Se (30 mg/L) in the culture medium significantly reduced the increase in OD. This could be due to the inhibitory effect of Se on the reproduction of yeast cells. After incubation of yeast in the presence of Se, the culture medium became slightly red in color, which indicates the reduction of selenite to red allotropes of Se (Se^0^)—the less toxic inorganic form of Se [22]. As reported by Fujs et al. [23], the presence of Se causes changes in yeast metabolic activity and expression of various genes. Oxidative stress then occurs [24], which causes membrane depolarization, i.e., a decrease in the electrical potential difference between the cellular cytosol and the culture environment [25]. To prevent this, yeast has developed several detoxification systems that protect cells in order to survive in adverse environmental conditions. It is believed that genes whose expression increases during stress conditions are also involved in the protection against Se stress. An example of such a process is increased biosynthesis of glutathione or antioxidant enzymes [26]. When encountering an excess of Se concentration, yeast cells activate the glutathione biosynthesis pathway to overcome the toxicity of this element. Glutathione is produced in the sulfur cycle of microorganisms, in which the main enzyme is sulfite reductase [27]. Glutathione plays an important role in protecting yeast against oxidative stress and has been shown to increase cell tolerance to Se.

**Figure 1:**
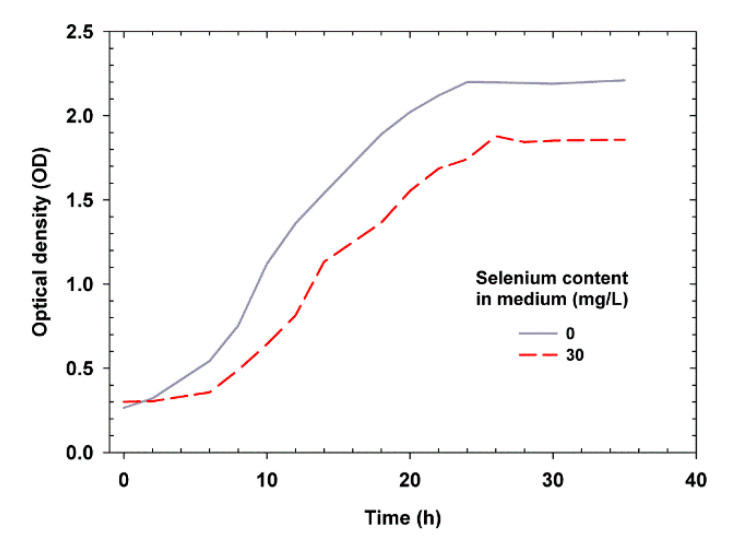
Effect of selenium on the growth of *Candida utilis* yeast cells.

The highest total increase in the OD of the yeast was observed in the YPD control medium after 24 h and in the medium containing Se after 26 h (Figure 1). Compared to the yeast grown in the medium with Se addition, the yeast grown in the control medium showed a fast growth rate. It should be noted that, during supplementation with Se, the yeast cells adapted to the new environment for a long time, and the duration of the adaptation phase (delay) was significantly longer (6 h) than that for the control medium (2 h). Substrate supplementation with Se at a concentration of 30 mg/L also caused a prolonged generation time. The consequence of these processes was a significant reduction of yeast budding processes in the logarithmic phase. The kinetic parameters of yeast growth are shown in Table 1.

The above findings confirm the results of previous studies reporting an inhibitory effect of Se on the growth of *C. utilis* ATCC 9950 (determined on the basis of cell biomass yield) in a medium containing 5% glycerol as the carbon source and waste potato water as the protein source [25]. The high concentration of Se in the culture medium significantly slowed down the growth of the tested yeast strains, as noted in this study. Estrada et al. [28] reported that *Lactobacillus* isolated from Mexican cheeses were sensitive to high levels of sodium selenite; the growth of the tested bacterial strains was inhibited at the concentration of 400 mg/L sodium selenite. Miletić et al. [29] observed a reduction in the growth of the fungus *Coriolus versicolor* due to the presence of Se. The studied fungi were subjected to a bioreactor culture in media with the addition of sodium selenite, and the inhibitory effect was already observed at the concentration of 10 mg Se^4+^/L. Similar relationships were also reported by Lusa et al. [30] who observed that the concentration of 6 mM sodium selenite reduces the growth rate of *Pseudomonas* sp. T5-6-I. The presence of Se induced a significant increase in the duration of the delay phase as compared to the control culture, which was also observed in our studies.

The presence of Se in the culture medium extended the time period for the cells to adapt to new unfavorable culture conditions. It was also confirmed that Se increased the mortality rate [30], which could mainly be associated with the occurrence of cell autolysis in the stationary growth phase. The primary importance in this phenomenon is attributed to the accumulation of metabolites (elemental form, Se^0^) by cells, resulting in the weakening of cytoplasmic membranes [16].

### 2.2. Effect of Se on the Activity of Antioxidant Enzymes

**Figure 2:**
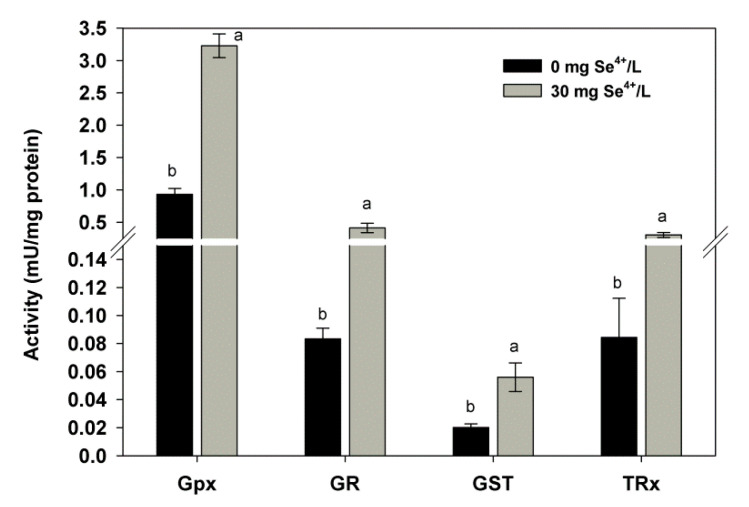
Effect of selenium on the activity of antioxidant enzymes in yeast biomass *C. utilis* (glutathione peroxidase, GPx; glutathione reductase, GR; glutathione S-transferase, GST; thioredoxin reductase, TRxR). Means with the same letter (a,b) did not differ significantly (acc. Tukey’s HSD test).

During evolution, organisms developed sophisticated antioxidant systems to maintain smooth cell function under adverse culture conditions. However, the relationship between Se and breeding stress has hardly been investigated. We observed that the presence of 30 mg/L of Se in the culture medium increased the activity of antioxidant enzymes (Figure 2) and found an increase in glutathione peroxidase (GPx) and glutathione S-transferase (GST) activities in Se-enriched biomass. Their values were 3.22 and 0.056 mU/mg, respectively. The obtained results are consistent with those of Assunção et al. [31] where the PGx content in yeast also increased as the concentration of Se in the growth medium increased. This finding agrees with a previous study which showed that a low concentration of Se increased the activity of antioxidant enzymes (including GPx, glutathione reductase (GR), and superoxide dismutase (SOD)), which consistently inhibited lipid peroxidation [25]. Wang et al. [32] studied *Gracilaria lemaneiformis* seaweed and confirmed the hypothesis that the increasing dose of Se resulted in increased activity of GPx. Therefore, these changes in enzyme activity can be considered as indicators of stress.

In the present study, the presence of 30 mg/L of Se increased GR activity by 50% in comparison to that of the control yeast. The activity of this enzyme, which is involved in the glutathione synthesis pathway, is controlled by trehalose concentration [33]. In addition, GR catalyzes the reduction of disulfide bond in oxidized glutathione (GSSG). The entire process is dependent on NADPH, which is very important for maintaining a pool of reduced glutathione in the cell (GSH) [31]. In our previous study on *C. utilis* kept in aqueous solutions supplemented with different doses of Se, we showed that the presence of Se increased the content of antioxidant enzymes and reduced glutathione [25]. The key strategy for yeast survival in the presence of Se is to control the dynamics of cytoplasmic membranes. If membranes are affected by changes in the content and composition of fatty acids, then they are more prone to attack by reactive oxygen species (ROS). Because of these processes, lipid peroxidation increases and changes occur in the morphological structure of cells. The consequence of such processes is a decrease in yeast viability. 

Thioredoxin reductase (TRxR) in yeast also increased its activity under the influence of Se. In comparison with the control sample, the content of this enzyme increased by as much as 72%. Such a large increase in the activity of this enzyme might be due to the result of the very low tolerance of *C. utilis* cultured in YPD medium to the presence of Se. TRxR is present in the mitochondria of most yeast and acts as a defense mechanism against ROS damage [34]. This enzyme is necessary to reduce ribonucleotides to deoxyribonucleotides, thereby contributing to DNA repair [35]. In addition, this enzyme is involved in the reduction of nonspecific linkages in protein structures that have undergone oxidation [25]. An excess of Se may reduce the intracellular pool of glutathione, causing the accumulation of ROS in yeast cell structures. Such processes can severely disrupt yeast metabolism through oxidative damage to cellular components. The production of ROS through enzymatic and nonenzymatic systems results in changes in the structure of fatty acids, causing lipid peroxidation, and thus forming lipid peroxides. However, the adaptation of yeast cells to stress conditions is very complex. Some mechanisms of stress response are still poorly understood, especially those associated with the adaptation of cells to the presence of various elements in the culture medium. Hence, it is very important to conduct further research on the effect of Se on the entire antioxidant system. Our results show that the cooperative action of the entire antioxidant enzyme system prevents the negative effect of Se on yeast cells. Based on the results obtained, we have shown similar responses of the tested antioxidant enzymes to the presence of Se.

### 2.3. Effect of Se on Trehalose and Glycogen Content

**Figure 3:**
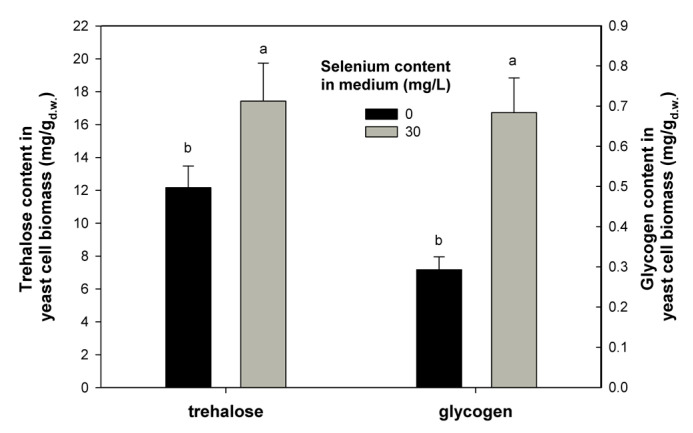
Effect of selenium on trehalose and glycogen content in *C. utilis* yeast cells. Means with the same letter (a,b) did not differ significantly (acc. Tukey’s HSD test).

The previously described antioxidant properties of Se-containing enzymes (including GPx and TRxR) are associated with the content of trehalose and glycogen in yeast cells. Diversified antioxidant protection in yeast resulting from the presence of Se is essential for the proper functioning of cells. We showed that the presence of 30 mg/L Se caused an increase in trehalose (17.43 mg/g) and glycogen (0.684 mg/g) content in the yeast biomass of *C. utilis* compared to that obtained from the control culture (Figure 3). The results obtained support the hypothesis that increased accumulation of trehalose and glycogen in cells might be associated with the presence of Se. The altered trehalose level proves that yeast cells were induced to synthesize trehalose in response to oxidative stress. Therefore, the increase in these metabolites is indicative of a protective effect against yeast cells. The task of the first sugar (trehalose) is to protect cell membranes after exposure of organisms to various environmental stresses [36].

Trehalose protects lipids and proteins against oxidation by changing the activity of individual biochemical reactions, which has an inhibitory effect on the generation of ROS [37]. According to Saharan and Sharma [33], trehalose functions in coordination with glutathione to control stress associated with free radicals in cells. Furthermore, Sharma et al. [38] demonstrated that the decrease in GSH with an increase in trehalose is associated with heat shock in both control and Se-supplemented cells. It is probably therefore a general adaptive reaction of yeast cells to proxidative stress caused by the presence of Se. Studies reported by Li et al. [39] have shown that yeast grown under stress caused increased accumulation of trehalose in their cellular structures. Glycogen is an intracellular polymer composed of glucose subunits, thus affects the normal growth of yeast by providing constant access to a carbon source [40]. It was found that, in yeast cells, glycogen degradation is associated with the production of sterols and unsaturated fatty acids, which are necessary to maintain cell vitality [41]. The production and use of carbon (glycogen) reserves in cells is also a common mechanism for yeast to adapt to adverse stress conditions [42]. According to the data presented by Possik and Pause [43], glycogen mediates hypoosmotic-anoxic stress resistance in the nematode *Caenorhabditis elegans*. As reported by Chen et al. [44], glycogen may play a protective role in yeast cells under osmotic stress when cell wall integrity is compromised. The oxidative stress resulting from the presence of Se causes cellular disorders in yeast cells, which cause changes in the functioning of individual organelles. Hence, the functioning of the entire antioxidative system in yeast is very important. It appears that Se induces various protection responses through increased accumulation of glycogen and trehalose in *C. utilis*. In conclusion, the way this defense mechanism works depends largely on the stress factor (Se) used to induce oxidative stress.

### 2.4. Se Bioaccumulation

Se can accumulate in the cells of various microorganisms in organic, inorganic, or elemental form or as their mixture. The functioning of different pathways of Se accumulation in yeast may additionally affect cell growth and final cell density (OD) in culture media [30]. There are several published studies on Se speciation in the yeast *S. cerevisiae*, which is widely used in human and animal supplementation [12,14]. The ability of Se to bind to the biomass of *S. cerevisiae* ATCC MYA-2200 and *C. utilis* ATCC 9950 was compared, and it was observed that yeasts of the genus *Candida* were more efficient in binding Se [15]. The total Se content of as high as 4000 µg/g has been reported [14]. We found that the biomass of *C. utilis* ATCC 9950 bound 1368 µg Se/g (Table 2).

Contrary to the data published for the commercially available *C. utilis* strains [14], the analyzed samples showed low content of the low-molecular-weight (LMW, water-soluble) species, which accounted for less than 10% of the total Se; the corresponding value for *Saccharomyces cerevisiae* is usually in the range of 12–18% [14]. The bioaccumulation of Se by various microorganisms has been widely studied by many authors. As reported by Miletić et al. [45], the medicinal fungus *Coriolus versicolor* grown in medium supplemented with 10 and 20 mg Se/L could accumulate 970 and 1300 µg/g of Se, respectively. Ullah et al. [46] showed that the probiotic bacteria *Bacillus subtilis* BSN313 grown in Se-enriched medium at the dose of 12 µg/mL could bind 2.123 µg Se/g. Egressy-Molnár et al. [47] studied the process of Se binding by *Hericium erinaceus* (lion’s mane mushroom). The fungus could bind 42.3 µg Se/g. An interesting course of changes in the bioaccumulation of Se in yeast biomass was presented by Zhang et al. [26,48]. The authors found that *C. utilis* CCTCC M 209298 could accumulate approximately 1010 µg Se/g after bioreactor culture in medium supplemented with 15 mg Se/L. It should be emphasized here that, although there are few studies on the binding of Se by *Candida* yeast, it can be concluded that the possibility of accumulation of this element is different from that in other species of microorganisms. It is worth noting that the yeast *C. utilis* belongs to the group of microorganisms that have the status of GRAS (Generally Recognized As Safe), i.e., safe for humans and animals [49]. Depending on the concentration and chemical form of Se, its uptake varies between yeast species and depends on the activity of membrane transporters and physiological conditions. However, little is known about Se transport, which is the first step in the metabolism of this element, i.e., processes involving reduction, methylation, and incorporation into proteins [50]. According to Estrada et al. [28], *Sel*A and *Sel*D genes are involved in the bioaccumulation of Se and its incorporation into proteins. McDermott et al. [51] showed that the transport of selenite (IV) into yeast cells is responsible for the monocarboxylic symport carboxy *Jen1p*. This transporter is located in the yeast cell membrane. Its function is to collect various monocarboxylic acids from the extracellular environment (e.g., pyruvic acid, acetic acid, and lactic acid). Jen1p operates on the principle of an import transporter using Na^+^ ions. The mechanism of transport of the Se (IV) ion through Jen1p is explained by the structural similarity between Se anions and monocarboxylic acid anions [52]. It is worth noting that these molecules have similar dissociation constants. Se uptake is also mediated by transporters with low affinity for phosphates (Pho87p, Pho90p, and Pho91p), while the other two transporters (Pho84p and Pho89p) have high affinity. The transport of Se and phosphate ions occurs through both high and low affinity channels, but with varying efficiency. In a low phosphate environment, a high affinity transporter such as Pho84p is a major contributor to the binding of selenate (IV) ions by yeast. During high phosphate concentration, the transport capacity of Se through the Pho84p channel is reduced. Under these conditions, low affinity proteins are responsible for transport. This is associated with reduced Se absorption and increased resistance of cells to toxic Se doses [53]. In addition, the vacuolar selenodiglutathione transporter YCF1 may be responsible for the increased accumulation of Se by yeast cells. This transporter is found in the yeast vacuole membrane and is one of the best-studied subfamily transporters ABCC [36]. The uptake of selenate by yeast can be mediated by sulfate transporters (Sul1p and Sul2p) due to the chemical similarity between selenate and sulfate [53]. Se is involved in the metabolism of sulfur in the yeast cell structures, and, as a part of many proteins, it may modify their conformational structure, leading to toxic effects and changes in functional activity. Thus, its accumulation impairs a wide range of cellular functions, which leads to a reduction in the rate of cellular metabolism, growth, and viability of yeast.

### 2.5. Se Speciation

The principal analytical approach to Se speciation, which is also used in this work, has been based on the fractionation of biological extracts by chromatography while specifically monitoring Se by inductively coupled plasma mass spectrometry (ICP-MS). The increase in the chromatographic resolution, the introduction of multidimensional separation approaches, and the growing sensitivity of ICP MS detection owing to collision cell and triple quadrupole mass spectrometers have led to the increasing number of unidentified peaks in HPLC–ICP MS chromatograms [54]. The absence of retention time standards has rendered electrospray MS indispensable for standardless identification of naturally synthesized Se compounds [54].

The widely approved method to characterize selenized yeasts to assess their “organic characteristic” is their SeMet content; it is a measure of the yeast quality with values reaching more than 60% in good quality preparations [55]. Its determination can be validated using the SELM-1 standard reference material (National Research Council of Canada), although the physicochemical properties of yeast produced by different methods may vary from the SELM-1 yeast [56]. SeMet content in the studied sample (Table 3) was relatively low (ca. 10%), which, however, corresponds to the previous findings on *Candida* yeasts (syn. *Torula*) [14,57]. The other selenoamino acid, selenocysteine, which accounts for less than 5% in *S. cerevisiae* in the studied sample, is present at the level of more than 20% of the total Se. These data allow estimating the incorporation of Se into yeast protein fraction at ca. 30% of the total Se present in the biomass. Se in the form of the amino acid selenocysteine (SeCys) is incorporated into the catalytic center in selenoproteins. These proteins are involved in the detoxification and capture processes of ROS.

**Figure 4:**
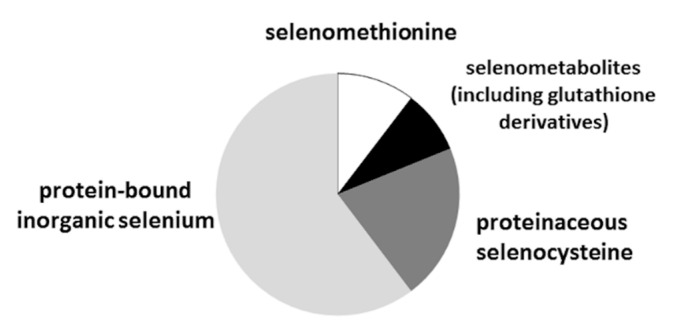
Percentage distribution of selenium among different selenium compounds groups.

**Figure 5:**
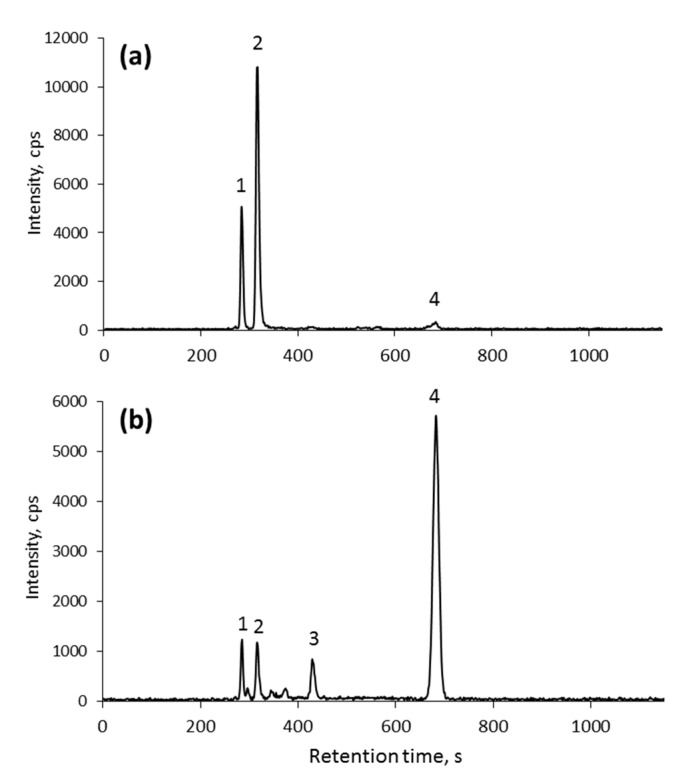
C8 RP HPLC-ICP MS chromatogram of the proteolytic digest of selenized yeast: (**a**) *C. utilis*; and (**b**) CRM SELM-1. Identification of the species based on spiking with standards of: (1) SeCys-CAM; (2) Se-CAM; (3) SeMet-CAM; and (4) SeMet.

The majority of Se (ca. 60%) is present in the form of inorganic Se as water-insoluble (mostly protein) fraction (Figure 4). This species is present in the chromatograms as peak 2 (Figure 5). The nature of the binding of inorganic Se remains to be fully elucidated; it is believed to be mostly bound to cell walls, although a contribution from selenite-binding proteins can also be expected. Their presence in many organisms has been well documented, and a binding site consisting of two neighboring Cys residues was identified [58]; such easily accessible sites are present in many yeast proteins and can scavenge (bind) inorganic Se present in Se-rich growth medium.

The speciation analysis of organoselenium metabolites obtained from the biomass of microorganisms depends on extraction conditions and on the stability of the extracted analytes [59]. The characterization of LMW Se (water-soluble species) led to the identification of five glutathione derivatives in the 450–700 Da mass range (Table 4). These species were already reported in *S. cerevisiae* [21,60]. In contrast with previous studies on commercially available *Candida* (syn. *Torula* yeast) supplements, no selenohomolanthionine has been detected [14].

**Figure 6:**
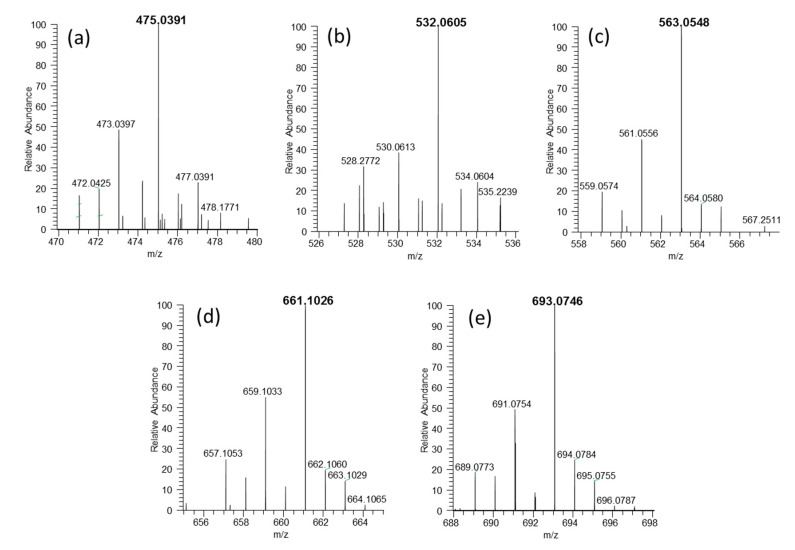
(**a**–**e**) ESI MS spectra showing isotopic pattern of Se-glutathione species detected in *C. utilis* water extract.

Se is present in the nature in the form of six natural isotopes, namely Se^74^, Se^76^, Se^77^, Se^78^, Se^80^, and Se^82^ with natural abundances of 0.86%, 9.23%, 7.60%, 23.69%, 49.80%, and 8.82%, respectively; this ratio can be observed in mass spectra of Se-containing species and is a basis of MS data mining for selenometabolites. The characteristic isotopic patterns resulting from the presence of an Se atom in the metabolites’ molecules are shown in Figure 6. In our previous study [61] using the UHPLC-ESI-Orbitrap-MS/MS method in *C. utilis* yeast biomass, we found the presence of Se compounds with mass from 195.9 to 376.9 *m*/*z*. Ward et al. [59] showed that the commercially available yeast contained Se compounds combined with glutathione, which corroborated the earlier findings of Arnaudquilhem et al. [21]. According to literature data [37], cell protection against oxidative processes predominantly occurs through the functioning of glutathione as a reducing agent. The antioxidant effect of this process may involve protecting the -SH groups against irreversible oxidation and thus against irreversible loss of biological activity of many enzymes [25].

### 2.6. Detection and Characterization of SeNPs

The production of SeNPs by microorganisms is a widespread phenomenon in nature, which has raised considerable interest in recent years [62]. In this context, the presence of Se-bearing NPs in the sample was investigated by single particle (SP)-ICP-MS and transmission electron microscopy (TEM). SeNPs have gained considerable interest recently owing to their antioxidant, antimicrobial, and anticancer properties [63].

**Figure 7:**
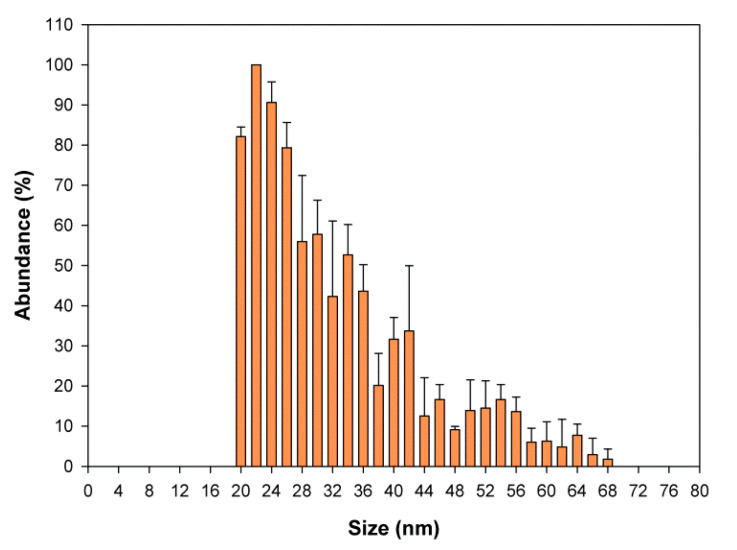
Size distribution of SeNPs.

**Figure 8:**
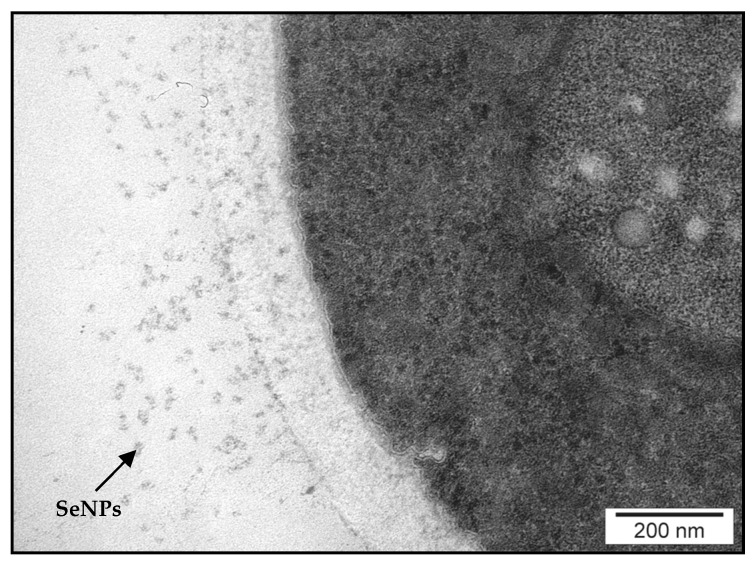
TEM image of SeNPs in yeast cell wall.

SP-ICP-MS measures the size distribution of metal-containing NPs in a single measurement [64]. The results obtained show the presence of SeNPs of 20–40-nm diameter (Figure 7). As the size limit of detection for SeNPs is around 20 nm [65], the occurrence of smaller nanoparticles could be missed. The presence of SeNPs in the yeast cell wall was confirmed by TEM (Figure 8). The differences in the sizes of NPs may result from the difference in Se oxidation [27], although the results obtained are in accordance with the findings of Hamza et al. [66]. The size of SeNPs obtained from the biomass of *Yarrowia lipolytica* NCIM 3589 ranged between 30 and 60 nm. Faramazi et al. [63] reported particle sizes of 75–709 nm in *S. cerevisiae*. Similar results were reported by Samant et al. [67]. TEM showed that the average particle size in the biomass of *Citrobacter freundii* KP6 was between 45 and 70 nm. For *Magnusiomyces ingens* LH-F1 (CGMCC No. 10367), the average particle size was 87.8 nm [62].

The formation of Se nanocolloids in cellular cytosol is associated with the detoxification process [16]. This is one of the mechanisms of protecting yeast cells against adverse culture conditions. Yeast reduces the soluble sodium selenite (Na_2_SeO_3_) to a red elemental form (Se^0^) [67]. According to Lian et al. [62], SeNPs may contain some proteins and lipids on their surface, which further stabilize their structure. The formation of SeNPs in yeast grown at different concentrations of this element causes a change in the color of the biomass of cells from bright yellow at the beginning of incubation to orange or even red. This finding confirms the result of our previous research where yeast biomass grown in high Se concentration (including 80 mg Se^4+^/L) showed a reddish coloration [68].

## 3. Materials and Methods 

### 3.1. Biological Material

The present study used the yeast strain *Candida utilis* ATCC 9950 obtained from the collection of pure cultures of the Department of Food Biotechnology and Microbiology, Warsaw University of Life Sciences-SGGW. Cellular biomass of the *C. utilis* strain was obtained after 24 h of shaking (SM-30 Control, E. Bühler, Bodelshausen, Germany), with a vibration amplitude of 200 cycles/min) in liquid YPD medium enriched with sodium selenite (IV). The active acidity of the substrates was set at 5.0. Media and aqueous solutions of Se salts (Na_2_SeO_3_) were sterilized at 121 °C for 20 min (Hirayama Autoclave HG80, Saitama, Japan). Then, a saline working solution was added to sterile YPD media in volumes such that the final Se content in the experimental media was 30 mg/L. The obtained yeast biomass after culture was centrifuged (3000× *g*, 10 min, 4 °C; Centrifuge 5804R, Eppendorf, Hamburg, Germany) and then washed twice with sterile distilled water. The obtained biomass was lyophilized and stored for further analysis.

### 3.2. Determination of Optical Density

Optical density (OD) was measured to determine the effect of Se on yeast cell growth. In brief, 270 µL of model (YPD) and experimental media (with the addition of Se^4+^ at 30 mg/L) and 30 µL of yeast from inoculation culture were added to all wells of the Bioscreen C apparatus plate (Oy Growth Curves Ab Ltd., Helsinki, Finland). Control samples (without the addition of biological material) were simultaneously prepared. Microcultures were performed for 35 h at 28 °C with continuous shaking. Measurement and registration of the change in the OD of yeast cells were performed automatically using a wideband filter with a wavelength of 420–580 nm. From the obtained yeast growth results, the minimum and maximum OD values in the logarithmic growth phase were determined. The correct growth rate ($\mu_{\max}$) was calculated using the formula: $\mu_{\max}=\left({\mathrm{lnOD}}_{max}-{\mathrm{lnOD}}_{min}\right)/\Delta t_{\log}$. The generation time (g) was determined from the formula: $g=\ln2/\mu_{\max}$. In addition, the total increase in the optical density of the culture (ΔOD) was determined.

### 3.3. Preparation of Cell Extract to Determine Trehalose, Glycogen and Antioxidant Enzyme Activity

After 24 h of yeast cultivation in control and Se-enriched culture media, the cell suspension was centrifuged (3000× *g*, 10 min, 4 °C) using a centrifuge (5804R, Eppendorf, Hamburg, Germany). The cell biomass was washed twice with 0.1 M phosphate buffer at pH 7.4. Yeast cells were disintegrated in a laboratory mill by using 0.3–0.5 mm glass beads at 4 °C. In the process of yeast cell disintegration, depending on the method used, test buffers provided with commercial biochemical kits were used in the mill. The cell extract obtained after disintegration (homogenate) was centrifuged (11,000× *g*, 5 min, 4 °C), and the obtained supernatant was transferred to new tubes, frozen at −80 °C, and used for spectrophotometric determinations.

### 3.4. Determination of Trehalose and Glycogen Content in Yeast Cell Biomass

The Trehalose Assay Kit (Megazyme, Warsaw, Poland) enzyme test was used to determine the trehalose content of the cellular biomass of *C. utilis* ATCC 9950. Measurements were performed at 340 nm. The content of trehalose in yeast cell biomass was expressed as milligrams of dry sugar substance per gram of yeast biomass.

The second test performed during the study was to determine the glycogen content of the cellular biomass of *C. utilis*. The Glycogen Assay Kit (BioVision, Mountain View, CA, USA) was used for this purpose. The absorbance of the supernatant was measured after incubating the samples in dark for 30 min at 570 nm. The glycogen content of yeast cell biomass was expressed as milligrams of dry sugar substance per gram of yeast biomass. Absorbance measurements were performed using a Multiskan SKY spectrophotometer (Thermo Scientific, Warsaw, Poland).

### 3.5. Biochemical Determination of Antioxidant Enzymes

Thioredoxin reductase (TrxR, EC 1.8.1.9) assay was performed using commercial kits (BioVision, Mountain View, CA, USA). Measurements were conducted at 412 nm and after incubation at 25 °C for 20 min. One unit of TrxR activity was defined as the amount of enzyme that produces 1 mole (TNB) in 1 min at 25 °C.

The amount of glutathione reductase (GR, EC 1.6.4.2) in extracts obtained after the yeast disintegration process was determined using enzyme kits of BioVision. GR corresponds to the amount of enzyme that catalyzes the conversion of 1 nmol of oxidized glutathione (GSSG) to its reduced form (GSH) in 1 min at 37 °C per milligram of protein (mU/mg).

The amount of glutathione peroxidase (GPx, EC 1.11.1.9) was determined using tests developed by BioVision. The absorbance of the cell extract was measured at 340 nm. The absorbance level decreases as a result of oxidation of NADPH to NADP+, which is directly proportional to the activity of GPx. One unit of GPx activity is the amount of enzyme that catalyzes the oxidation of 1 μmol glutathione by cumene hydroperoxide in 1 min per milligram of protein (mU/mg) at 25 °C.

Glutathione S-transferase (GST, EC 2.5.1.18) activity was determined in extracts obtained after yeast disintegration by using monochlorobimane (MCB) as a substrate (BioVision). Absorbance measurements were performed at 380 nm excitation wavelength and 460 nm emission wavelength. One unit of GST activity corresponded to the amount of enzyme that catalyzed the conversion of 1 nmol of substrate into product within 1 min per milligram of protein (mU/mg).

### 3.6. Determination of Protein Content

Protein concentration in the supernatants was determined according to the method of Kieliszek et al. [69]. The standard curve was prepared using bovine albumin (Sigma-Aldrich, Warsaw, Poland). Absorbance measurements of all enzymatic experiments were performed using a Multiskan SKY spectrophotometer (Thermo Scientific).

### 3.7. Yeast Observation Under an Electron Microscope

The centrifuged yeast biomass (3000× *g*, 10 min, 4 °C) was fixed in 2.5% glutaraldehyde at 4 °C for 2 h. The fixing process was performed in a 1% solution of osmium tetroxide for 1 h at 4 °C. After dehydration, the yeast biomass was embedded in EPON 812 epoxide and left for 24 h. The material was then incubated at 60 °C for 48 h. Blocks were cut using an ultramicrotome (LKB, Bromma, Sweden). Ultrathin sections with the biological material were contrasted with 9% uranyl acetate and 0.5% lead citrate. The obtained cell samples and SeNPs were observed under a transmission electron microscope (JEM 1220 TEM, JEOL, Tokyo, Japan).

### 3.8. Total Se Determination

In this test, 0.2 g of sample was accurately weighed in a DigiPrep tube and left overnight with 2.5 mL of HNO_3._ Then, 1 mL of H_2_O_2_ was added, and the sample was digested in a DigiPrep digestion system (digestion program: 0–30 min up to 65 °C, 30–240 min at 65 °C). The digests were diluted to reach HNO_3_ concentration of 4% and analyzed by ICP-MS using the optimized conditions. The quantification was performed by the method of standard addition at 4 levels. The samples were analyzed in triplicate. The analytical blanks and SELM-1 were analyzed in parallel.

### 3.9. Determination of Total Selenomethionine (SeMet)

A 0.2-g sample was incubated with 5 mL of a protease XIV solution (20 mg protease in 30 mM TRIS buffer, pH 7.5). Three consecutive incubations (17 h at temperature 37 °C) with fresh portions of the enzyme solution were carried out. After each incubation, the sample was centrifuged (4000× *g*, 4 °C, 10 min), in a 5804R centrifuge (Eppendorf, Hamburg, Germany) and the supernatant was transferred to a separate vial to which 5 μL of β-mercaptoethanol were added. Upon the completion of the whole series, the three supernatants were pooled together and analyzed by anion exchange HPLC-ICP MS in gradient elution mode. Buffer A was 20 mM acetic acid–10 mM triethylamine (pH 4.7) and Buffer B was 200 mM acetic acid–100 mM triethylamine (pH 4.7). The program was: 0–5 min: 0% B, 5–30 min: 0–100% B, 30–40 min. The quantification was carried out by the method of standard additions with SeMet at four levels. The samples were analyzed in triplicate. The analytical blanks were included in the measurements. SELM-1 was analyzed in parallel.

### 3.10. Determination of Protein Selenocysteine (SeCys) and Selenomethionine (SeMet)

A 0.2-g sample was leached 3 times with a fresh portion 5 mL of water (by sonication during 1 h and centrifugation at 4000× *g*) in a 5804R centrifuge (Eppendorf, Hamburg, Germany); the solution after each washing was discarded. Two milliliters of 0.1 M TRIS buffer (pH 7.5) were added to the residue followed by addition of 30 µL of dithiothreitol (DTT) solution (0.2 M solution in 0.1 M TRIS buffer, pH 7.5) and 50 µL of iodoacetamide (IAM, 0.5 M solution in TRIS buffer). Then, a fresh 150 μL aliquot of the DTT solution was added and the mixture was shaken for 1 h in order to destroy excess of IAM. Subsequently, 10 mL of TRIS buffer an aliquot of 1 mL of a protease solution (30 mg protease XIV in 2 mL of 100 mM TRIS buffer, pH 7.5) were added and the sample. After 2-h incubation, a second aliquot of 1 mL protease solution was added and the sample was incubated overnight at 37 °C. After centrifugation, the supernatant was removed, filtrated on 2-kDa filter, and analyzed by HPLC-ICP MS. An Agilent 1200 HPLC system (Agilent, Tokyo, Japan) used as a delivery system for RP was coupled with an Agilent 7700 ICP-MS instrument (Agilent, Tokyo, Japan) fitted with Pt cones and a 1 mm i.d. injector torch using chromatographic conditions given in Table 5. The quantitation of SeCys and SeMet was carried out using the method of standard (SeCys-CAM and SeMet, respectively) additions at three levels. The samples were analyzed in duplicate. The analytical blanks were measured in parallel.

### 3.11. Identification of Se-Compounds in Water Fraction by ESI MS

Water fraction obtained in point 3.10 was analyzed by ESI MS after filtration with 2 kDa cutoff filter. HPLC separation was carried out using gradient elution (Table 5) on C18 reverse phase column connected to a Dionex system (Ultimate 3000RS) and detection was performed on an Orbitrap Fusion Lumos Tribrid Mass Spectrometer (Thermo Scientific, France, www.thermofisher.com). The instrument was operated in the positive ionization mode at a resolution of 240,000. The electrospray voltage was set to 3.5 kV, the ion transfer tube temperature to 350 °C, and the vaporizer temperature to 450 °C. The sheath gas, auxiliary gas, and the sweep gas were set to 60, 15 and 2 units, respectively.

### 3.12. Sample Preparation for SeNP

Enzymatic digestion. The digestion/extraction procedure included four steps:

1. Two hundred milligrams of a Se-rich yeast sample were suspended in 5 mL of water, bath sonicated for 1 h in A Branson B2510 ultrasonic bath (Branson, Danbury, CT, USA), and centrifuged at 4500× *g* for 10 min in a 5804R centrifuge (Eppendorf, Hamburg, Germany).
2. The pellet was resuspended with a solution of 5 mL of 4% (*m*/*v*) Driselase (Sigma Aldrich, Saint-Quentin Fallavier, France) in 30 mM Tris (Sigma Aldrich, Saint-Quentin Fallavier, France) at pH 7.5, incubated at 25 °C for 17 h, and centrifuged at 4500× *g* for 10 min.
3. The pellet was resuspended with a solution of 5 mL of 4 mg/L protease (Sigma Aldrich, Saint-Quentin Fallavier, France) in 30 mM Tris at pH 7.5, incubated at 37° C for 17 h, and centrifuged at 4500× *g* for 10 min.
4. The pellet was resuspended with a solution of 5 mL of 4% (*m*/*v*) sodium dodecyl sulphate (SDS, Sigma Aldrich, Saint-Quentin Fallavier, France), bath sonicated for 1 h, and centrifuged at 4500× *g* for 10 min.

The supernatant was recovered and kept at 4 °C until analysis. An Agilent 7900 Inductively Coupled Plasma Mass Spectrometer (ICPMS) was used for detection and characterization of SeNP. The sample introduction system consisted of a concentric nebulizer and a quartz cyclonic spray chamber. Isotope ^80^Se was monitored with a dwell time of 100 µs and a total acquisition time of 60 s. The settling time was set to 0 during analyses. H_2_ was used as reaction cell gas at a flow rate of 5 mL/min to remove background interferences according to the method developed by Jiménez-Lamana et al. [65]. Single Nanoparticle Application Module for ICP MS MassHunter software (Agilent Technologies, Santa Clara, CA, USA) was used for data processing.

## 4. Conclusions

Se was found to negatively affect the growth of the yeast strain *C. utilis* ATCC 9950. The activity of antioxidant enzymes and trehalose and glycogen content increased in the presence of Se, which indicated an increased activity of the antioxidant system in the yeast. The obtained results suggest that the tested yeast was not tolerant to the used concentration of 30 mg Se^4+^/L. This was associated with stress conditions and detoxification processes conducted by cells. The consequence of this, among others, was the formation of SeNPs, which conferred a slightly red color to yeast biomass. High-molecular-weight Se compounds containing glutathione in their structures were observed. The formation of these compounds indicates the conditions of oxidative stress. The identification of other metabolic biochemical pathways of Se should be considered to discover the origin of the observed organic Se compounds. The obtained test results can serve as a link that connects the effect of Se metabolic transformations and progressive detoxification processes in yeast cells. They are likely to provide information on the biochemical reaction mechanisms and the possibilities of their regulation. It may be possible to obtain strains with increased tolerance to Se and stress. Finally, it should be noted that it is very important to properly control dietary supplements enriched with Se and to determine optimal breeding conditions that would not adversely affect the performance of the final product. Appropriate monitoring of the effect of this element and examination of yeast physiology and the occurrence of stress on the cells will help to control the entire production process.

## Figures and Tables

**Table 1:** Parameters characterizing the growth *Candida utilis* ATCC 9950 during culturing in the control YPD medium and experimental media enriched in selenium.

Selenium Content in the Medium	Δt_lag_ (h)	Δt_log_ (h)	OD_min lag_	OD_max log_	µ_max_ (h^−1^)	G (h)	ΔOD
0 mg/L	2	22	0.54	2.2	0.063	10.09	1.934
30 mg/L	6	20	0.49	1.9	0.067	10.29	1.578

**Table 2:** Total selenium determination and speciation in *Candida* yeast.

Sample	Fraction	Selenium (µg/g)	RDS (%)
control	total	5.0 ± 0.12 ^c^	2.4
	water soluble	2.0 ± 0.13 ^d^	6.4
Se-enriched	total	1368 ± 69.36 ^a^	5.1
	water soluble	113 ± 2.67 ^b^	2.4

^a–d^ Means with the same letter did not differ significantly (acc. Tukey’s HSD test).

**Table 3:** Results of selenium speciation analysis of *Candida* yeast.

Sample	Selenium Compounds					
	Selenomethionine (SeMet, µg/g)	RSD (%)	Selenocysteine (SeCys, µg/g)	RSD (%)	Selenium Inorganic (Se IV, µg/g)	RSD (%)
SELM-1	1162 ± 45.5	3.9	102 ± 7.6	7.4	132 ± 8.5	6.5
*Candida utilis*	138 ± 4.6	3.4	279 ± 18.1	6.5	804 ± 45.4	5.6

**Table 4:** Selenium species identified in the water soluble fraction of *Candida* yeast.

Nr	Formula	Exp. Mass	Error (µg/g)	Name Compounds	Name
a	C_13_H_23_O_8_N_4_SSe^+^	475.0387	−1.9	selenoglutathione-cysteine	188609-44-1 or 117135-55-4 *
b	C_15_H_26_O_9_N_5_SSe^+^	532.0599	−2.3	glutathione-selenocysteinylglycine	1357479-87-8
c	C_16_H_27_O_11_N_4_SSe^+^	563.0544	−2.2	glutathione-2,3-DHP-selenocysteine	1006377-09-8
d	C_20_H_33_O_12_N_6_SSe^+^	661.1022	−2.3	selenoglutathione-glutathione	161973-63-3
e	C_20_H_33_O_12_N_6_S_2_Se^+^	693.0746	−1.6	selenodiglutathione	1052197-78-0

* Two CAS numbers are available depending of the R, S configuration.

**Table 5:** Chromatographic Conditions Used.

Column	Eluent	Gradient Elution	Temperature (°C)	Flow Rate (mL/min)	Sample Volume (µL)	Detection
C8 Alltima 4.6 × 250 mm	A: 0.1% HFBA * in water B: 0.1% HFBA in MeOH **	0–15 min 3% B 15–18 min 3-40% B 18–21 min 40% B 21–23 min 40-3 % B 23–30 min 3% B	10	0.9	10	ICP MS
C18 Zorbax Eclipse XBD 4.6 × 150 mm	A: 0.1% FA *** in water B: 0.1% FA in MeOH	0–2.5 min 3% B 2.5–15 min 3-50% B 15–20 min 50% B 20–20.5 min 40-3 % B 20.5–30 min 3% B	40	1.0	10	ESI MS

* heptafluorobutyric acid; ** methanol; *** formic acid.
